# Drought Stress Affects the Response of Italian Local Tomato (*Solanum lycopersicum* L.) Varieties in a Genotype-Dependent Manner

**DOI:** 10.3390/plants8090336

**Published:** 2019-09-07

**Authors:** Veronica Conti, Lavinia Mareri, Claudia Faleri, Massimo Nepi, Marco Romi, Giampiero Cai, Claudio Cantini

**Affiliations:** 1 Department of Life Sciences, University of Siena, Siena, SI 53100, Italy (C.F.) (M.N.) (M.R.) (G.C.); 2 CNR-IBE (Consiglio Nazionale delle Ricerche-Istituto per la Bioeconomia), Follonica, GR 58022, Italy

**Keywords:** tomato, Italian varieties, drought stress, oxidative stress, physiological response

## Abstract

Drought stress is one of the most severe conditions for plants, especially in the face of the emerging problem of global warming. This issue is important when considering economically relevant crops, including the tomato. For these plants, a promising solution is the valorization of local agrobiodiversity as a source of genetic variability. In this paper we investigated how six Italian tomato varieties react to a prolonged period of water depletion. We used a multidisciplinary approach, from genetics to plant physiology and cytology, to provide a detailed overview of the response of plants to stress. The varieties analyzed, each characterized by a specific genetic profile, showed a genotype-specific response with the variety ‘Fragola’ being the most resistant and the variety ‘Pisanello’ the most susceptible. For all the parameters evaluated, ‘Fragola’ performed in a manner comparable to that of control plants. On the contrary, ‘Pisanello’ appeared to be more affected and showed an increase in the number of stomata and a drastic increase in antioxidants, a symptom of acute oxidative stress. Our work suggests the existence of a valuable reservoir of genetic biodiversity with more drought-tolerant tomato genotypes opening the way to further exploitation and use of local germplasm in breeding programs.

## 1. Introduction

The most common environmental factor affecting plant growth and productivity/yield is the lack or scarcity of water, a stressful condition known as “drought stress” [1]. By altering the water balance of cells and tissues, drought stress negatively affects many physiological and cellular processes, by acting both at the molecular level (e.g., by inducing protein denaturation), at the cellular level (cell collapse), and at the level of the entire plant (wilting). Drought stress is a critical current problem and its study is progressively taking on a major role; given the increased frequency of acute drought conditions due to global warming, its severity will most likely be exacerbated by future climate change [2].

Plants have developed physiological, morphological, cytologic, and biochemical responses to avoid and/or limit the consequences of drought stress [3]. From a physiological point of view, the first response of plants to severe water deficit is stomata closure aimed to prevent the transpiration water loss [4]. In addition to stomata closure, plants can also reduce stomata size in response to prolonged water lack and can alter their number, length, and width [5]. In this regard, studies have shown an increase in stomatal density under conditions of moderate drought stress, and a decrease in stomatal density following prolonged periods of stress [6]. However, changes in stomata number and morphology under water stress condition are dependent on the species. Despite these species-dependent responses, in all plants stomatal closure is regulated by the ABA (abscisic acid) hormone with both a hydropassive and a hydroactive way [5]. One of the main consequences of stomata closure is the decrease in the inflow of CO_2_ and the concomitant increase of O_2_ concentration into the leaves and the consequent accumulation of photosynthesis-derived electrons, which can potentially react with molecular oxygen leading to the formation of ROS (Reactive Oxygen Species). Free ROS, if abundant and accumulating for a prolonged period, can react with biological structures, damage DNA, promote protein oxidation and cause lipid peroxidation [7]. Plants respond to oxidative stress by synthesizing specific enzymes able to scavenge ROS [8] and/or to produce antioxidant molecules such as polyphenols [9]. A further mechanism of tolerance to water stress is represented by compatible solutes, which allow a fine osmotic regulation thereby maintaining turgor pressure even in stressful conditions. Osmolytes such as ammonium-based compounds (polyamines, glycine betaine, and others), or derived from sugars (trehalose, mannitol, sorbitol, and others) or amino acids (proline) are reported to perform important functions in osmotic regulation against drought stress [10].

Tomato (*Solanum lycopersicum* L.) is a major crop grown all over the world as it is an important dietary source of vitamins (A and C), and carotenoids such as lycopene [11]. However, despite its large-scale cultivation, it is susceptible to abiotic and biotic stresses [12,13]. Both vegetative and reproductive processes of modern tomato cultivars can be severely compromised by drought stress, which inhibits seed development, reduces vegetative growth and compromises reproduction [14,15]. During vegetative growth, drought-treated tomato plants show a reduction in biomass production associated with a decrease of leaf production and a smaller plant height compared to control plants. Not only a reduced number of leaves but also a reduction in leaf area can decrease the photosynthesis yield [16]. In this regard, Pervez et al., [16] showed that tomato seedlings are sensitive to drought with a strong inhibition of light harvesting efficiency, photochemical conversion, and photosynthetic electron transport. Concerning the effect of water deficit on tomato reproduction, it is well known that drought stress during seed development decreases seed yield by shortening the final seed filling periods. In addition to this, as proved by the vigor test, drought stress occurring during seed formation or filling resulted in reduced seedling vigor and germination [16]. Other studies reported that drought stress significantly affected tomato yield, however increasing the irrigation state under a specific level did not positively influence the tomato yield which remains stable [17,18,19]. In addition to yield, lack of water also influences the volume, diameter, and composition of fruits (i.e., lycopene and total soluble sugars) [20]. All these parameters are useful indicators of drought resistance/susceptibility.

The observed susceptibility to drought stress needs the development of more resistant varieties able to cope with water stress while maintaining yield [21]. In this view, genetic biodiversity can be considered as a valuable resource for the enhancement of crop yield, for the resistance to diseases and pests, and for quality improvement. It is well accepted that a wider varietal and species diversity would enable agricultural systems to support productivity over a wide range of conditions, including stressful conditions. As a signatory country of the Convention on Biological Diversity (CBD) (Rio de Janeiro, 1992), Italy has promoted a national strategy for the conservation of biodiversity. In this regard, the Tuscany region has enacted a specific law (LR 64/04) to promote the protection of biodiversity and to recover local varieties at risk of extinction by classifying and including them in a regional repertoire (Regional Bank of Germplasm). The assessment of diversity in plants is routinely performed using several techniques. In the postgenomic era, molecular markers have emerged as powerful tools for the analysis of genetic variation. Simple sequence repeats (SSRs), also known as microsatellites, are repeats of up to 100 times of simple sequences of 1–8 base pairs [22]. They are highly polymorphic, abundant, present in both coding and non-coding regions, and co-dominantly inherited giving them a high power of discrimination [23]. SSRs have been widely employed to exploit genetic diversity in different plant species [24,25,26] including tomato [27,28,29].

The present work aimed to study the effects of drought stress in six local varieties of Tuscan tomato (*Solanum lycopersicum* L.). Such varieties, registered in the Regional Bank of Germplasm, are characterized by different morphological and agronomic characteristics. Tomato plants were genetically characterized using a set of selected SSR markers previously adopted for successful variety identification [27,28,29]. In addition to this, the varieties were physiologically evaluated using different parameters ranging from photosynthetic activity to stem diameter and plant height. To have a more complete analysis, we investigated other parameters such as stomata density, leaf morphology, and antioxidant activity. The work provides information on how locally adapted tomato varieties cope with water scarcity and it paves the way to further studies aimed to improve the tomato crop by using plant genetic diversity.

## 2. Results and Discussion

**Figure 1:**
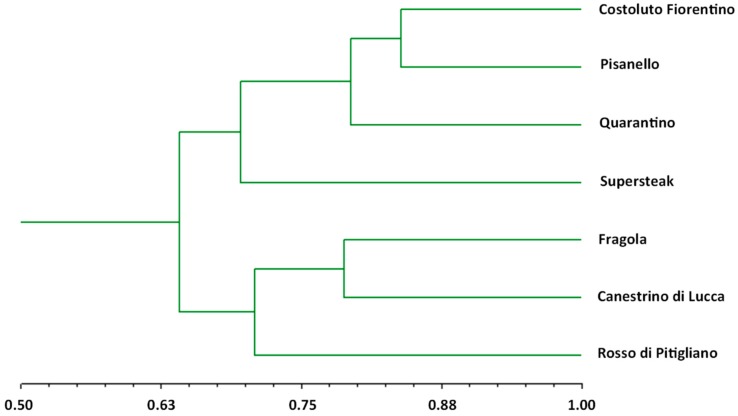
Hierarchical clustering (UPGMA algorithm) of six local varieties and one commercial cultivar (‘Supersteak’).

Genetic characterization of the six local varieties was performed using a set of 16 SSR markers selected from the literature [27,28,29]. Ten out of the 16 selected SSRs were polymorphic. Monomorphic markers, namely LEat016, LEcag003, LEatt001, LEcag001, LEcag001, and SSR603, were thus excluded from the analysis. The allelic profile of each variety was used to obtain a phylogenetic tree through the unweighted pair-group method for arithmetic averages (UPGMA) hierarchical clustering (Figure 1). The dendrogram shows that the method used was able to discriminate the tested varieties. In fact, the phylogenetic tree grouped the six varieties and the commercial variety into two major clusters, one consisting of ‘Costoluto Fiorentino’, ‘Pisanello’, ‘Quarantino’ and ‘Supersteak’ and the other one consisting of ‘Fragola’, ‘Canestrino di Lucca’ and ‘Rosso di Pitigliano’. In the first cluster, the commercial ‘Supersteak’ is genetically more distant to the other genotypes; similarly, ‘Rosso di Pitigliano’ is more distant to the other genotypes included in the second cluster. In the latter ‘Costoluto Fiorentino’ and ‘Pisanello’ are the most similar varieties. As reported by the Regional Bank of the Germplasm, the distribution of the above mentioned varieties is widely diffused in Tuscany and the cultivation of some varieties (e.g., ‘Canestrino di Lucca’), originally restricted to a specific area, has been expanded to the whole region. Therefore, it is not easy to explain the differences observed in the dendrogram considering the different origins of each variety. ‘Rosso di Pitigliano’ is the only one whose origin (South Tuscany) matches its distribution and its genetic profile supports this evidence.

In order to evaluate how drought stress can compromise the physiology of tomato plants, we monitored a series of physiological parameters related to plant growth such as growth index, stem diameter and leaf area, as well as other parameters directly or indirectly related to photosynthesis, such as photosynthesis efficiency, performance index, and stomata number; finally, we measured other parameters related to oxidative stress (which is a consequence of drought) such as superoxide distribution and content of antioxidant molecules. The physiological measurements were used to select the most resistant and the most susceptible variety for which we performed all the above-mentioned analyses. Supplementary Figures S1–S4 show the phenotype of plants at t_0_ (before treatment), t_1_ (half duration of stress), and t_2_ (end of stress). As can be seen, at t_1_ no appreciable differences were visible while at t_2_ it was clear there was a different degree of drought resistance among genotypes. Particularly, ‘Fragola’ seemed to be not affected and its phenotype was comparable to control (well irrigated plants). A lower level of resistance was exhibited by ‘Canestrino di Lucca’ and the commercial cultivar ‘Supersteak’ which were only partially damaged: leaves were green but lightly wilted with the edge of the lamina curved downward. More compromised appeared to be ‘Costoluto Fiorentino’ and ‘Rosso di Pitigliano’ with severely wilted and partially shriveled leaves. Such phenotype was exasperated in ‘Pisanello’ in which leaves were totally shriveled. In the middle scale there was ‘Quarantino’ whose phenotype was mildly affected, showing partially wilted leaves.

**Figure 2:**
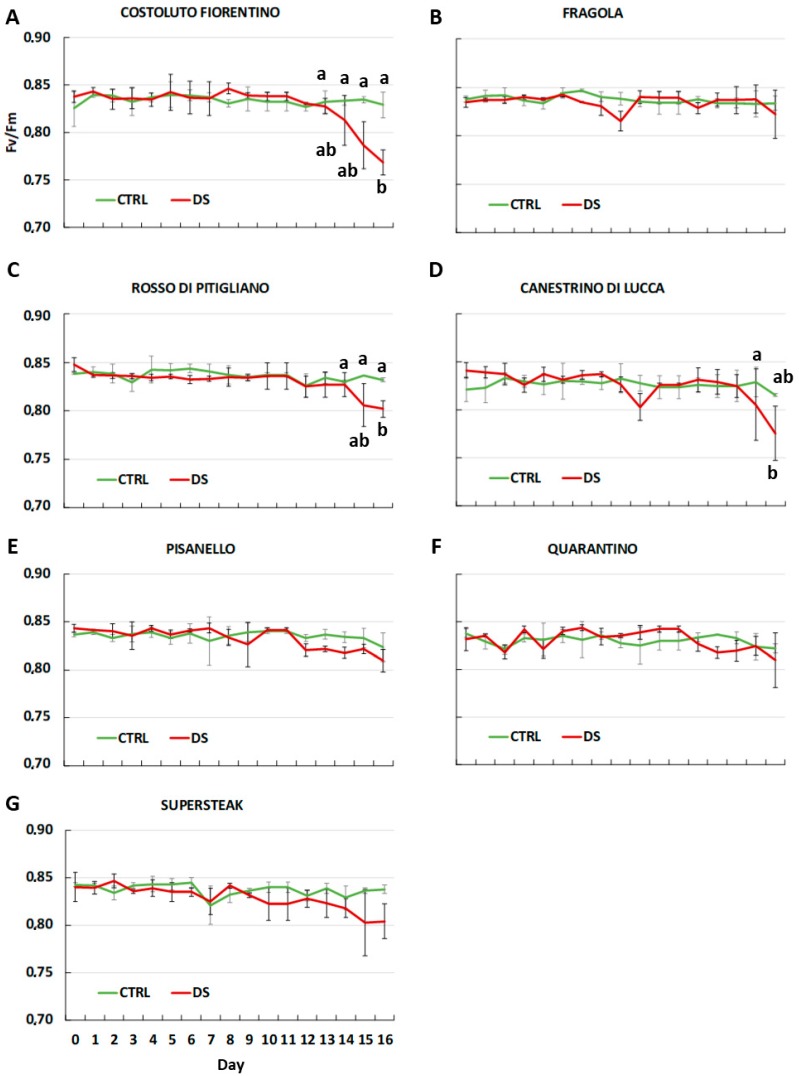
The maximum quantum yield of photosystem II (F_v_/F_m_) in six local varieties (**A**–**F**) and one commercial cultivar ‘Supersteak’ (**G**) of tomato (*Solanum lycopersicum* L.) subjected to drought stress (no water for 16 days) relative to the controls (regularly well-watered plants). Two-way factorial ANOVA followed by Tukey post-hoc test was used to determine the statistical significance of differences. Points with the same lower-case letters do not differ significantly (*p* > 0.05).

Photosynthetic efficiency is one of the processes most affected by drought stress. Particularly, as reported for other abiotic stresses, lack of water causes a serious impairment of the photosynthetic machinery with photosystem II (PSII) being one of the most susceptible components [30]. Photosynthetic efficiency can be evaluated by F_v_/F_m_ measurement that is the maximum quantum yield of PSII. The results here obtained showed that F_v_/F_m_ did not change during the first eight days of stress (Figure 2) with no differences between control and stressed plants. Changes became clear in the later stages of stress. Particularly, ‘Costoluto Fiorentino’, ‘Rosso di Pitigliano’, ‘Canestrino di Lucca’ showed a significant decrease in photosynthetic efficiency starting from day 12–13, with a clear decline to day 16. Among the local varieties, ‘Pisanello’ started a slight decline in F_v_/F_m_ value from day 10 and it remained constant until day 16. Interestingly, the values of F_v_/F_m_ of the commercial cultivar ‘Supersteak’ decreased after day 8, in contrast to local varieties. The only two exceptions were ‘Quarantino’ and ‘Fragola’ for which F_v_/F_m_ measurements remained similar in both well-watered and stressed plants for the whole period of stress. Such results show that they did not suffer serious damages even at day 16. Similar to our results, Paknejad et al., (2007) [31] showed that in wheat, different irrigation treatments negatively influenced F_v_/F_m_ and they also described a correlation between grain yield and fluorescence paramenters (i.e., F_v_/F_m_): such parameters can be thus considered as reliable characteristics to adopt for evaluation of genotypic differences in drought stress tolerance.

**Figure 3:**
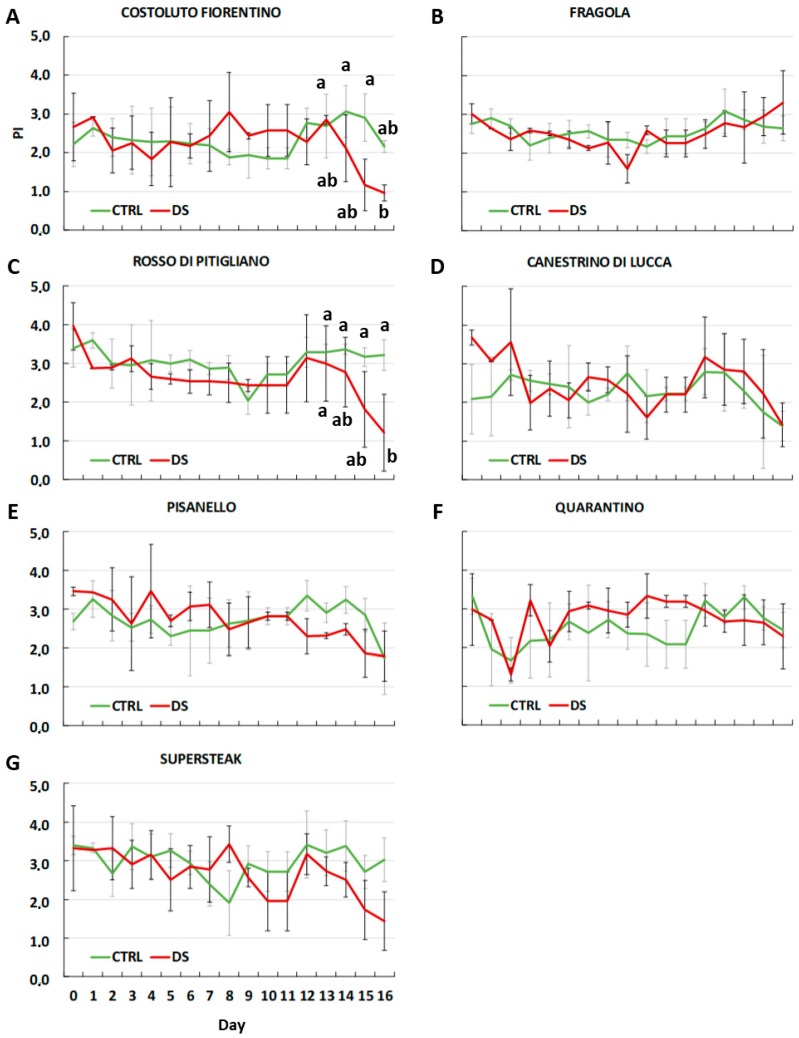
Performance index (PI_ABS_) in six local varieties (**A**–**F**) and one commercial cultivar ‘Supersteak’ (**G**) of tomato (*Solanum lycopersicum* L.) subjected to drought stress (no water for 16 days) relative to the controls (regularly well-watered plants). Two-way factorial ANOVA followed by Tukey post-hoc test was used to determine the statistical significance of differences. Points with the same lower-case letters do not differ significantly (*p* > 0.05).

The results of the Performance Index (PI) (Figure 3) were more variable during stress treatment, but they confirm the F_v_/F_m_ values. In fact, ‘Costoluto Fiorentino’, ‘Rosso di Pitigliano’ ‘Canestrino di Lucca’, ‘Pisanello,’ and the commercial ‘Supersteak’ showed a slight decrease in PI already from day 12–13 with a sharp drop at day 16. On the contrary, for ‘Quarantino’ and ‘Fragola’ the trend was similar between well-watered and treated plants. These results show that among the tested varieties the process of photosynthesis is not equally affected by drought stress.

**Figure 4:**
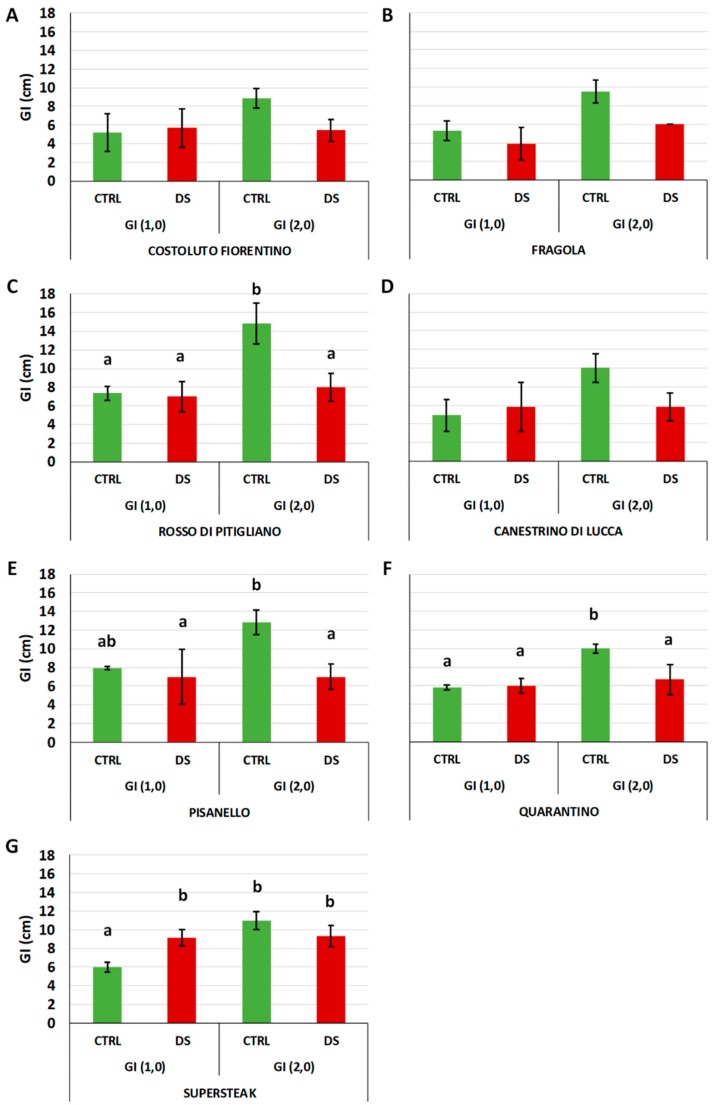
Growth Index (GI) in six local varieties (**A**–**F**) and one commercial cultivar ‘Supersteak’ (**G**) of tomato (*Solanum lycopersicum* L.) subjected to drought stress (no water for 16 days) relative to the controls (regularly well-watered plants). Two-way factorial ANOVA followed by Tukey post-hoc test was used to determine the statistical significance of differences. Points with the same lower-case letters do not differ significantly (*p* > 0.05). GI (_1,0_) = (h_1_ − h_0_)/2; GI (_2,0_) = (h_2_ − h_0_)/2.

Another physiological parameter symptomatic of stress-induced damaged is plant growth. This latter, herein expressed as growth index (GI), is linked to carbohydrate resources and energy generated by photosynthesis: stress exposure leads to over-excitation of the photosynthetic electron transport chain, alters photosystem activity determining a reduction in carbohydrate synthesis and energy needed for plant growth [32]. With a trend similar to the one reported for F_v_/F_m_, the results of GI_1,0_ in all the studied varieties did not show significant differences between control and stressed plants with the only exception being the commercial ‘Supersteak’ for which the GI_1,0_ value significantly increased. In contrast to that observed for GI_1,0_, GI_2,0_ was severely compromised for all stressed plants. For all varieties, including the commercial genotype, a sharp drop was recorded, with ‘Rosso di Pitigliano’ and ‘Pisanello’ being the most damaged varieties (Figure 4). Our results are in agreement with other studies that reported a reduction in plant growth under water deficit. In this regard, Wahb-Allah et al., 2007 showed that tomato plant height was inhibited by decreasing the amount of irrigation water. Interestingly and similar to our results, authors also described a genotype-dependent effect with some genotypes less affected than others [17].

**Figure 5:**
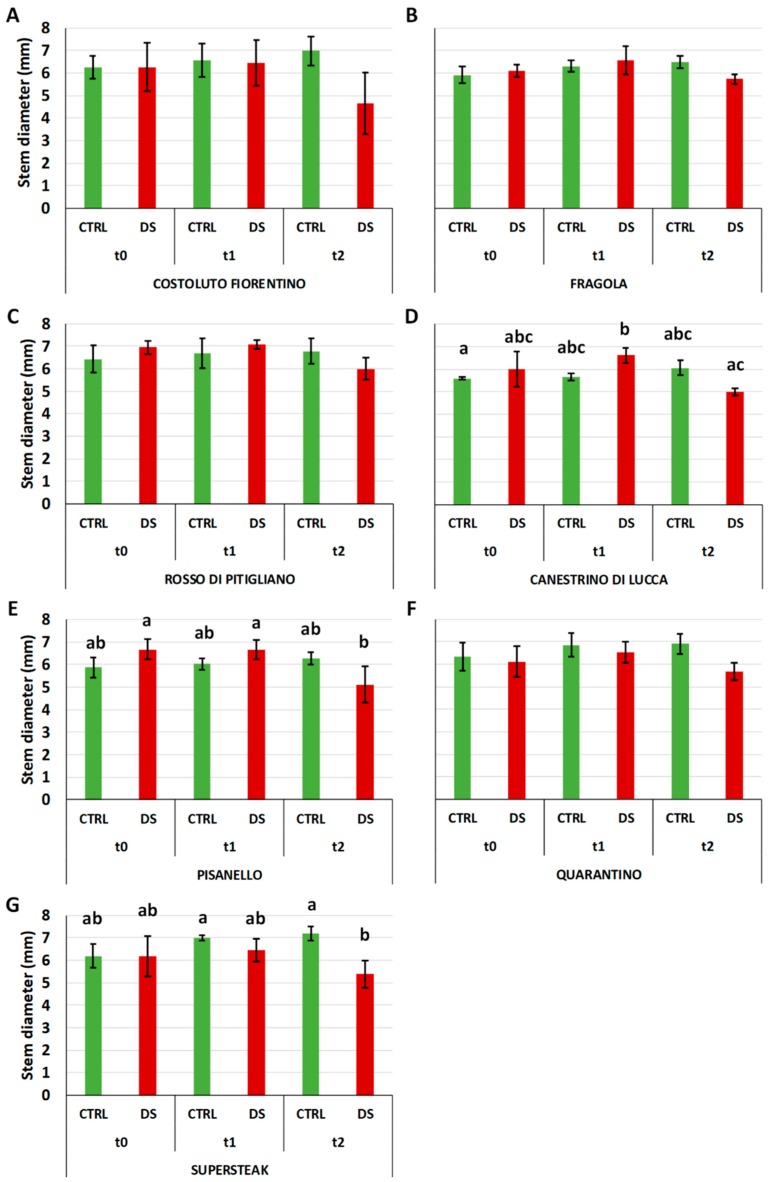
Stem diameter in six local varieties (**A**–**F**) and one commercial cultivar ‘Supersteak’ (**G**) of tomato (*Solanum lycopersicum* L.) subjected to drought stress (no water for 16 days) relative to the controls (regularly well-watered plants). Two-way factorial ANOVA followed by Tukey post-hoc test was used to determine the statistical significance of differences. Points with the same lower-case letters do not differ significantly (*p* > 0.05).

While GI is indicative of stress, stem diameter cannot be considered a discriminating parameter. In this regard, the results at t_0_ and t_1_ (Figure 5) showed that well-irrigated and stressed varieties were still similar while at t_2_ a slight drop in diameter for all varieties was observed.

Overall, these results together with the phenotypic evaluation allowed us to define the most resistant variety (′Fragola′) and the most susceptible (′Pisanello′) on which to focus the analyses which follow. To highlight differences with commercial cultivars, we also included the ‘Supersteak’ cultivar in the analysis.

**Figure 6:**
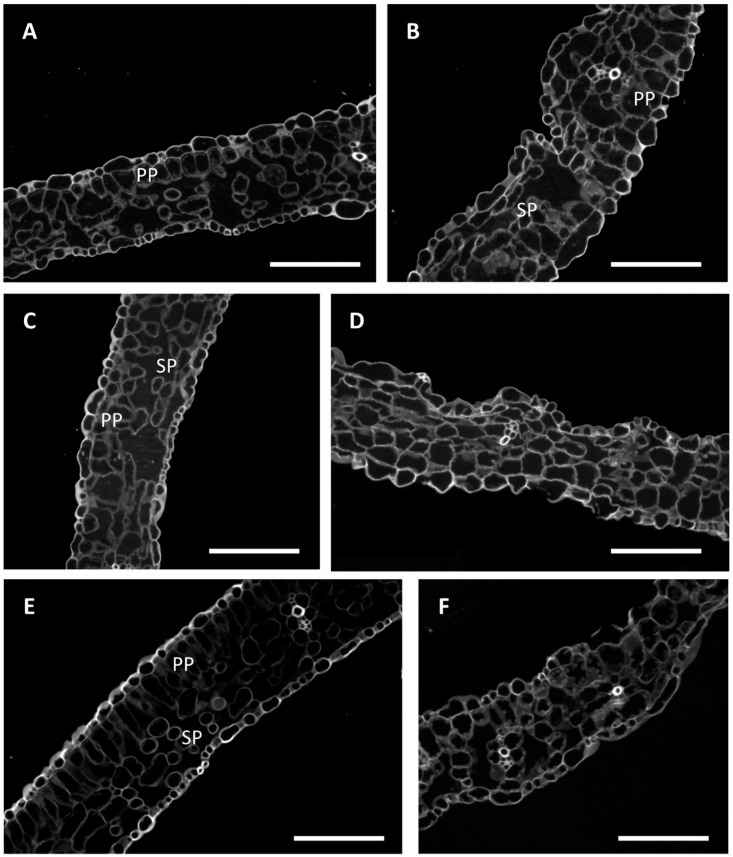
Calcofluor White (CFW) staining in leaves of two local varieties (**A,B**—‘Fragola’; **C**,**D**—‘Pisanello’) and one commercial cultivar (**E**,**F**—‘Supersteak’). **A**,**C**,**E** are the controls (regularly well-watered plants) and **B**,**D**,**F** are the stressed (no water for 16 days). PP, palisade parenchyma; SP, spongy parenchyma. Scale bar: 100 µm.

First, a morphological analysis of leaf structure was performed at t_2_ to understand if the impairment of photosynthesis efficiency could be associated with a cytological alteration of leaf tissues. In agreement with the above reported results indicating ‘Fragola’ (Figure 6A,B) as the most resistant genotype, cellulose staining showed that the general structure of leaves was maintained under drought stress condition: it was still possible to distinguish the palisade parenchyma (PP) and cells retained their typical shape. However, slight damage was detected at the level of spongy parenchyma (SP), with cells approaching each other and leaving few intercellular spaces. A more severe alteration of leaf cytological structure was observed in ‘Pisanello’ (Figure 6C,D) where it was no longer possible to distinguish between palisade parenchyma and a spongy one and cells no longer had a well-defined shape and were remarkably close to each other. Similar to what was observed for ‘Pisanello’, ‘Supersteak’ (Figure 6 E,F) showed a general disorganization of leaf structure: stressed plants exhibited only partially the palisade parenchyma with cells very close to each other, causing the loss of the typical structure of spongy parenchyma, as opposed to that found in the control where palisade cells are well organized, and the spongy parenchyma shows the typical intercellular spaces. Our results agree with that reported for other plants such as olive [33] and blackberry [34] in which a profound rearrangement of leaf architecture has been described. In olive, the authors reported a decrease of the size of both epidermal and mesophyll cells and a corresponding reduction in the mesophyll intercellular space volume that could be interpreted as a strategy to block the movement of water vapor. Zhang et al., 2017 [34] showed that in blackberry the total leaf transections and the upper and lower leaf epidermis declined due to continuous water deficit while the cell walls of upper and lower epidermis and the spongy tissue cell walls were clearly thickened: such thickness of the leaf cuticle was interpreted as a clear indication of drought tolerance.

**Figure 7:**
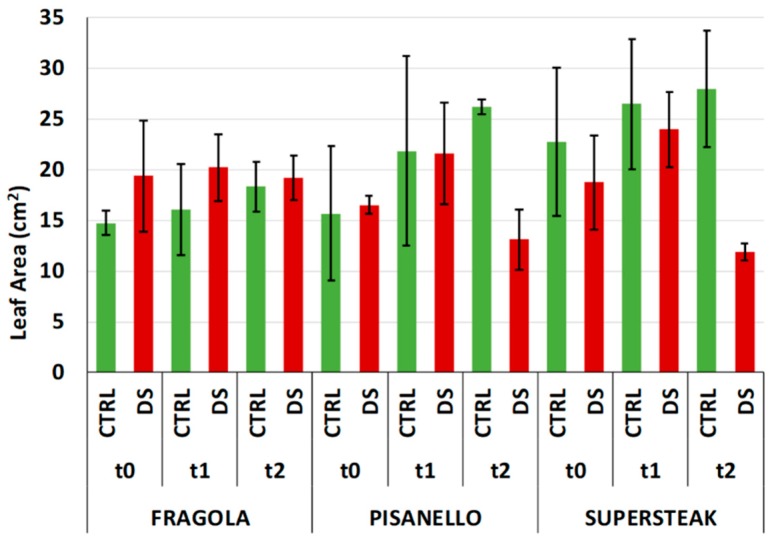
Leaf Area in two local varieties (‘Fragola’ and ‘Pisanello’) and one commercial cultivar ‘Supersteak’ of tomato (*Solanum lycopersicum* L.) subjected to drought stress (no water for 16 days) relative to the controls (regularly well-watered plants). Two-way factorial ANOVA followed by Tukey post-hoc test was used to determine the statistical significance of differences. Points with the same lower-case letters do not differ significantly (*p* > 0.05). The p-values for ‘Pisanello’ and ‘Supersteak’ are near to significance, respectively *p* = 0.07 and *p* = 0.08. t_0_ = before the start of treatment, t_1_ = half duration of the stress, t_2_ = the end of the stress.

**Figure 8:**
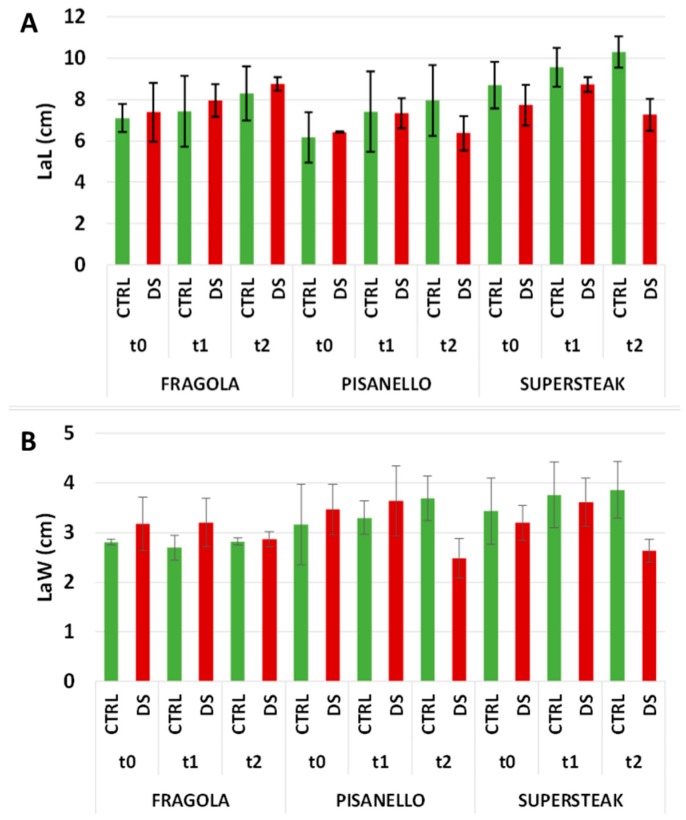
(**A**) LaL (Lamina Length) and (**B**) LaW (Lamina Width) in two local varieties (‘Fragola’ and ‘Pisanello’) and one commercial cultivar ‘Supersteak’ of tomato (*Solanum lycopersicum* L.) subjected to drought stress (no water for 16 days) relative to the controls (regularly well-watered plants). Two- way factorial ANOVA followed by Tukey post-hoc test was used to determine the statistical significance of differences. Points with the same lower-case letters do not differ significantly (*p* > 0.05). t_0_ = before the start of treatment, t_1_ = half duration of the stress, t_2_ = the end of the stress.

In addition to analyzing leaf morphology, we also measured other parameters related to the leaf including leaf area (LA), lamina length (LaL), and lamina width (LaW). As shown in Figure 7, at t_0_ and t_1_ the leaf areas of well-irrigated and stressed plants were comparable for all three varieties. At t_2_, some differences could be appreciated; in particular the leaf area of ‘Pisanello’ and ‘Supersteak’ varieties decreased in comparison to well irrigated plants although the p values for the differences are slightly above the significance level (*p* = 0.07 and *p* = 0.08 respectively). The ‘Fragola’ variety, on the contrary, was not particularly damaged and, consequently, the leaf area remained similar for both stressed and control plants. The results of leaf morphology were further confirmed by the length of the LaL and leaf width LaW. As for LaL (Figure 8A) and LaW (Figure 8B), these measurements did not show differences at both t_0_ and t_1_ between control and stressed plants, suggesting that in the middle of the stress period (t_1_) the tomato varieties did not show severe damage as regards morphometric measurements of leaves. At t_2_, both LaL and LaW were severely reduced in ‘Pisanello’. Conversely, in ‘Fragola’, for which the photosynthetic process was not damaged by the lack of water, leaf area and related parameters were not affected. Based on these results, we hypothesize that the ‘Fragola’ variety succeeds in carrying out the photosynthetic process even in the presence of reduced availability of water. Our results agree with Liu and Stützel, 2004 [35] who reported a reduction in leaf area and leaf dry mass ratio. The same authors also describe a simultaneous increase in root dry mass ratio indicating that under water deficiency plants invest energy in the enhancement of root system in order to absorb water more efficiently. Similar results were also reported for Populus [36], soybean [37], and other species [4].

**Figure 9:**
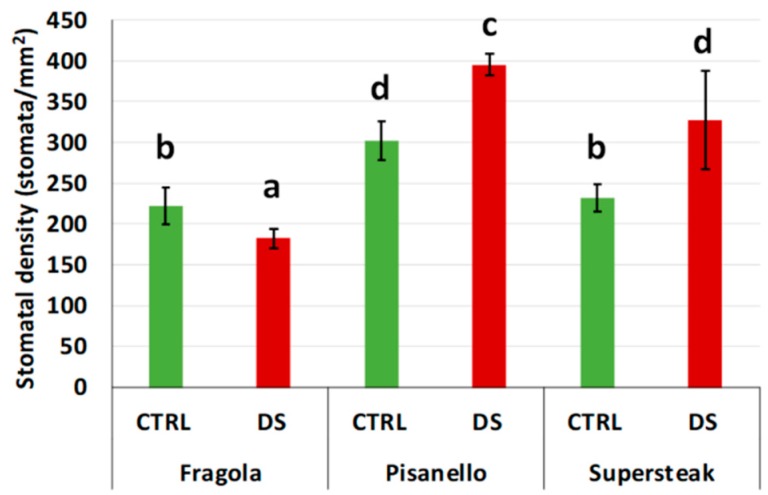
Stomatal density in two local varieties (‘Fragola’ and ‘Pisanello’) and one commercial cultivar ‘Supersteak’ of tomato (*Solanum lycopersicum* L.) subjected to drought stress (no water for 16 days) relative to the controls (regularly well-watered plants). Two-way factorial ANOVA followed by Tukey post-hoc test was used to determine the statistical significance of differences. Points with the same lower-case letters do not differ significantly (*p* > 0.05).

Another response that plants can put in place to response to drought stress is the regulation of stomata number and activity [5]. In the case of drought stress, plants close the stomata to limit water loss due to evapotranspiration. Even the stomata number and size are affected by stress: increasing the intensity of water stress generally increases the stomata density and reduces the size of the stomata [6]. These modifications have been interpreted as adaptations to drought as the increase in stomata number also increases the supply of CO_2_ to leaves while size reduction limits the loss of water. In the present manuscript, the ‘Pisanello’ and the commercial genotype ‘Supersteak’ increase the stomata density as a response to stress, while the ‘Fragola’ variety decreased stomata density in respect to well-watered plants suggesting that ‘Fragola’ does not perceive the lack of water as stress (Figure 9). Another possible explanation is that a reduction in stomata density implies a limitation of water loss. Our results are in agreement with a recent study in rice showing that transgenic plants with a reduced stomatal density (and a consequent low stomatal conductance) were more able to conserve water. The authors also reported that such plants had equivalent or even higher yields under water lack condition and they concluded that a reduced stomata density is a useful trait, to have plants that better perform in the climate change scenario [38].

**Figure 10:**
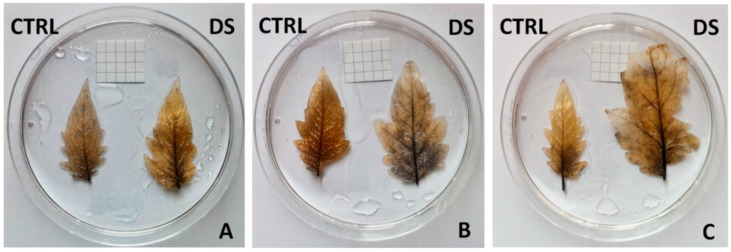
Nitro Blue Tetrazolium (NBT) staining in leaves of two local varieties (**A**—’Fragola’; **B**—’Pisanello’) and one commercial cultivar (**C**—’Supersteak’). On the left are the controls (regularly well-watered plants) and on the right are the stressed samples (no water for 16 days). The side of the square in the background is 0.5 mm.

As mentioned above, the closure of stomata in response to stress can lead to a decrease in CO_2_ and an increase of O_2_, thus to an imbalance of electrons in the photosynthetic system with the consequent formation of reactive oxygen species (ROS) [9]. The increase in ROS amount can lead to oxidative stress, capable of damaging various cellular structures [7]. In response, cells put in place scavenging mechanisms to reduce the extent of damage [10]. In the present work, we investigated the distribution of superoxide radicals in the leaves of selected varieties. As shown in Figure 10 the superoxide radicals visible as blue/purple after Nitro Blue Tetrazolium (NBT) staining were found especially in the vascular bundles or in their proximity. ‘Fragola’ (Figure 10A) and ‘Supersteak’ (Figure 10C) do not accumulate superoxide radicals under drought stress: only a less intense staining is visible close to the principal leaf vein. On the contrary, in ‘Pisanello’ (Figure 10B) superoxide radicals are more widely distributed in the leaf: NBT staining not only labeled the veins but also the leaf basal lamina.

**Figure 11:**
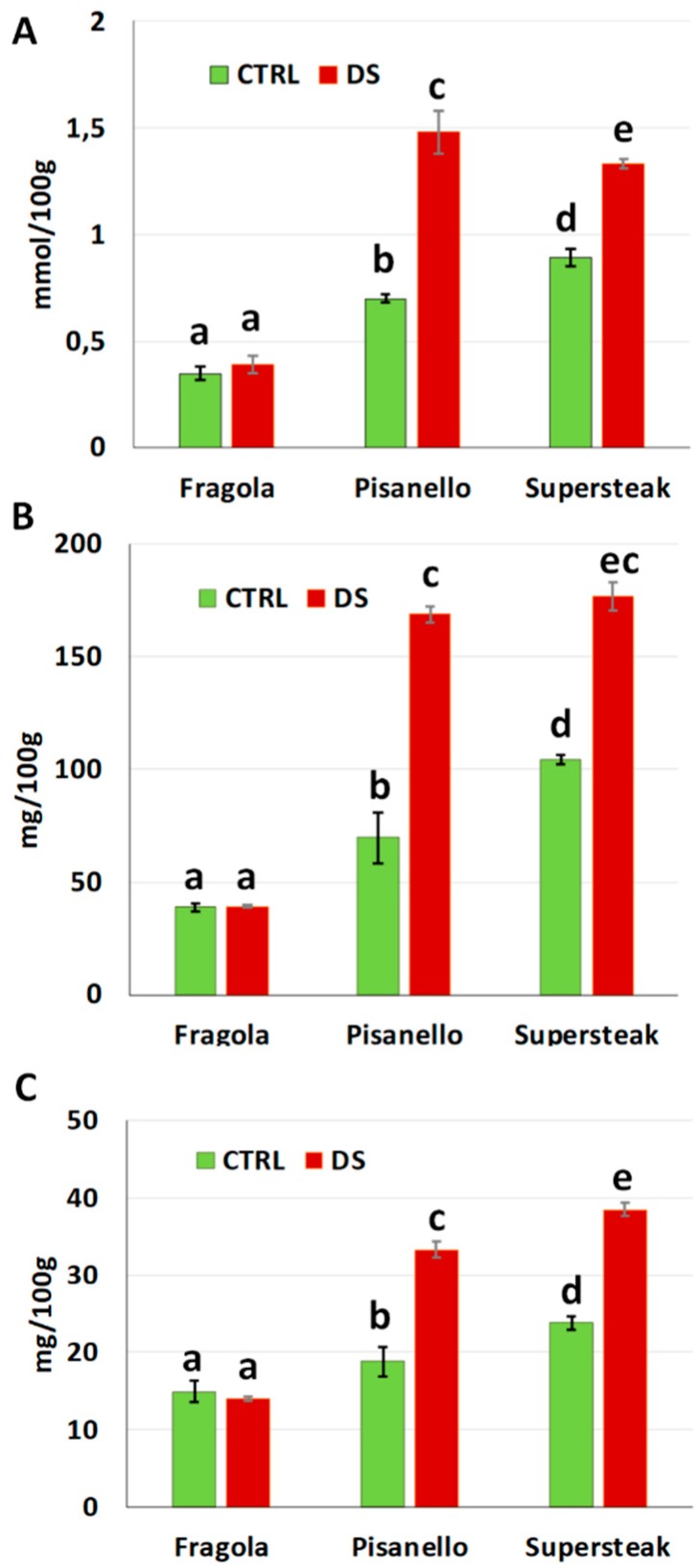
(**A**) total antioxidant content, (**B**) polyphenols content and (**C**) flavonoids content of two local varieties (‘Fragola’ and ‘Pisanello’) and one commercial cultivar (‘Supersteak’) of tomato (*Solanum lycopersicum* L.) subjected to drought stress (no water for 16 days) relative to the controls (regularly well-watered plants). Two-way factorial ANOVA followed by Tukey post-hoc test was used to determine the statistical significance of differences. Points with the same lower-case letters do not differ significantly (*p* > 0.05).

The production of reactive oxygen species can induce as response the synthesis of antioxidant molecules. Figure 11A reports the total antioxidant power, expressed as mmol/100 g. ‘Fragola’ did not increase the antioxidant capacity, which remained at the same level as the control. On the other hand, ‘Supersteak’ and ‘Pisanello’ greatly increased the antioxidant ability even more, a symptom of a response to oxidative stress. These data were also confirmed by the total content of polyphenols and flavonoids expressed as mg/100g (Figure 10B,C). Once again, stressed plants of ‘Fragola’ were comparable to control while in the stressed plants of ‘Pisanello’ and ‘Supersteak’ the content of polyphenols and flavonoids increased. We can, therefore, assume that the ‘Fragola’ variety does not undergo oxidative stress, at least not as substantially as to require the production of antioxidants. Our results are in agreement with other papers that report an increment in polyphenols and flavonols under drought stress condition. For example, in tea such an increase has been viewed as a possible marker of drought resistance where resistant clones showed a high content of polyphenols with a low content of water in the soil [39]. In addition to this, the increase would be useful as a strategy to enhance the nutritional and bioactive properties of plants as suggested for *Amaranthus* [40].

## 3. Materials and Methods

### 3.1. Plant Material

Seeds of six tomato local varieties namely: ‘Costoluto Fiorentino’, ‘Fragola’, ‘Rosso di Pitigliano’, ‘Canestrino di Lucca’, ‘Pisanello’, and ‘Quarantino’ were retrieved from the Regional Bank of the Germplasm, while seeds of the commercial cultivar ‘Supersteak’ (producer company: Royal Seeds^®^ s.r.l., Mirandola, Italy) were purchased at large retailers. ‘Supersteak’ was chosen among the many commercial tomato cultivars because it is a vigorous and robust plant which is also resistant to common soil diseases.

### 3.2. Genetic Characterization of Tomato Local Varieties by SSRs

Genetic characterization was performed for all the listed varieties. Genomic DNA was isolated from 100 mg of frozen young leaves using the DNeasy^®^ Plant Mini Kit (QIAGEN^®^, Hilden, Germany). DNA extracted from tomato leaves was used to amplify a set of 16 SSR markers selected from the literature (Table S1). PCR assays were performed using the protocols reported in the literature [27,28,29]. All PCR reactions were carried out using Eppendorf Mastercycler^®^ epGradient. Each PCR reaction was performed in a final volume of 25 μL containing: 1 U of DreamTaq DNA Polymerase (ThermoFisher, Waltham, MA, USA), 0.5 μL 10 mm dNTP mix (ThermoFisher, Waltham, Massachusetts, USA), 0.25 μmol/L of each primer (Sigma-Aldrich, Saint Louis, MO, USA), 1× DreamTaq reaction buffer (ThermoFisher, Waltham, MA, USA), and 30 ng of genomic DNA. The PCR amplification program was: 94 °C for 2 min (DNA denaturation), 40 cycles with 94 °C for 45 s (denaturation); Ta for 45 s (annealing) where Ta changes for each primer pair; 72 °C for 45 s (extension) and a final extension at 72 °C for 7 min. After amplification, PCR products were analyzed by electrophoresis on 1.8% agarose gel run with 1× TBE (89 mmol/L Tris borate, 89 mmol/L boric acid, 2 mmol/L EDTA) at 80 V.

### 3.3. Growth Conditions and Stress Treatment

Seeds of each variety were first germinated in Petri dishes with filter paper soaked with distilled water at a constant temperature of 25 °C in the dark. Afterwards, the seedlings were transferred to a cultivation chamber in a tray with wells (each well 4 × 5 × 6 cm) at a constant temperature of 25 °C with a 14 h/10 h light/darkness photoperiod with a PPFD (photosynthetic photon-flux density) of 350 μmol m^−2^ s^−1^ and with relative humidity of 50 ± 10%. Then two-leaf seedlings were moved into pots larger than 10 × 10 × 12 cm at the same growth conditions previously described. The substrate used for repotting operations was the VIGOR PLANT^®^ GROWING MEDIUM, Professional Mix. All plants were well-watered until the beginning of the water stress treatment maintaining water availability close to the capacity of the potting mix. The water-stress treatment began when plants were 30/40 cm high corresponding to 45 days after germination and was maintained for 16 days while the control group (CTRL) was kept in full irrigated regime. The duration of drought treatment was chosen in agreement with Landi et al., 2016 and Sánchez-Rodríguez et al., 2010 [41,42]. For each genotype, measurements were done on three control plants vs. three drought-stressed plants.

### 3.4. Determination of the Efficiency of Photosynthesis (F_v_/F_m_ and PI)

Photosynthetic efficiency was evaluated by using the Fluorometer Handy PEA 2000 (Hansatech Instruments King’s Lynn, Norfolk, UK). For each genotype, the values of F_v_/F_m_ and PI were collected daily for 16 days to identify the time when plants begin to perceive water stress. The following equations were used to calculate F_v_/F_m_ (1) and PI (2) parameters [43]:

F_v_/F_m_ = (F_m_ − F_0_)/F_m_ (1)

(2) $${\mathrm{PI}}_{\mathrm{ABS}}\;=\;\frac{1-\left(\frac{F_{0}}{F_{m}}\right)}{M_{0}/V_{j}}\times \frac{F_{m}-F_{0}}{F_{0}}\times \frac{1-V_{j}}{V_{j}}$$

where F_m_ is the maximum fluorescence value, F_0_ is fluorescence value at zero instant, F_v_ is the difference between F_m_ and F_0_, V_J_ is relative F_v_, and M_0_ is the initial slope of fluorescence kinetics. F_v_/F_m_, therefore, is an index from the maximum value of 1.00, equivalent to 100% of the maximum photochemical efficiency of photosystem II. Performance index (PI), a more sensitive parameter showing variations of the entire photosynthetic apparatus, including photosystem I (PSI) and II (PSII). F_v_/F_m_ and PI were measured for each genotype (1–7) in 3 plants over 16 days. Results are expressed as the average of the three replicas ± the standard deviation.

### 3.5. Measurement of Stem Diameter and Plant Growth

For each genotype, measures of stem diameter and growth were performed at time t_0_ (before the start of treatment), at time t_1_ (half duration of the stress—8 days without water) and at time t_2_ (at the end of the stress). Stem diameter at the bottom of plant was measured via a digital gauge (Powerfix^®^, Neckarsulm, Germany) and plant height, used for the estimation of growth index (GI) (3), was measured by a meter stick. The formula used to calculate GI was:

GI_f,i_ = h_f_ − h_i_/2 (3)

where h_f_ is final height (at time t_1_ or time t_2_) and hi is initial height (at time t_0_). Thus, this was summarized as GI_1,0_ the Growth Index between t_1_ and t_0_, GI_2,0_ that between t_2_ and t_0_. Height and stem diameter were measured for each genotype (1–7) in 3 plants in three timing points. For the height the Growth Index (GI) was calculated and the standard deviation was then computed by the error propagation. Results are expressed as the average of the three replicas ± the standard deviation.

### 3.6. Morphometric and Histological Evaluation of Leaves

The morphometric parameters used to evaluate leaves were leaf area (LA), lamina length (LaL), and lamina width (LaW). All these parameters were calculated for each variety to the same timing points (t_0_, t_1_, t_2_). At each interval, photos were taken of the leaves at the same developmental stage. Photos were then examined by ImageJ (National Institutes of Health, Bethesda, MD, USA) to determine the LA, LaL, and LaW. LA, LaL, and LaW were measured in 3 plants at three timing points. Results are expressed as the average of the three replicates ± the standard deviation.

At t_2_, overall leaf structure was analyzed by CFW (Calcofluor White) staining [44]. Leaves were taken at the same developmental stage and cut transversely at the midpoint. Samples were fixed in 2.5% glutaraldehyde (*w*/*v*) in 0.1 M phosphate buffer (pH 7.2) for 2 h at room temperature. After that samples were dehydrated in increasing ethanol concentration (10%, 30%, 50%, 70%, 90%, 100%) and then embedded in Technovit 7100 (Kultzer GmbH, Hanau, Germany). Semi-thin sections (4–5 µm) were obtained with Ultratome NOVA microtome (LKB, Bromma, Sweden). The resulting sections were stained with a 0.1% CFW solution diluted in distilled water for about 30 min. Slides were washed quickly with distilled water, coated with tin foil to avoid fluorescence decay, and finally put in the stove at 37 °C for 5 min to evaporate the excess water. Finally, samples were examined with a fluorescence microscope (Imager. Z1 Apotome Zeiss, Oberkochen, Germany).

### 3.7. Stomatal Density

Stomata density was calculated using the protocol proposed by Xu and Zhou (2008) [6]. Briefly, leaves from each variety, at the time t_2_, were sampled at the same stage of development. A thin layer of transparent nail polish was uniformly applied on the lower surface of leaves and, once dried, it was pulled away. The resulting molds were then examined under the optical microscope (Axiophot Zeiss, Oberkochen, Germany). For each sample, 10 photos were taken, and stomata were counted using ImageJ (National Institutes of Health, Bethesda, MD, USA). Stomata density was expressed as the stomata number per leaf area unit (in mm^2^). Stomata number was measured for each genotype in 3 plants at the end of stress, totaling 10 photos for each genotype. Results were expressed as the average of the 10 counts ± the standard deviation.

### 3.8. Determination of Superoxide Radicals

Superoxide radicals in the leaves at t_2_ (after 16 days of drought) were detected by NBT (Nitro Blue Tetrazolium) staining [45]. For each variety leaves at the same stage of development were analyzed. They were incubated in the NBT staining solution (0.5 mg/mL) for 8 h at room temperature in darkness and then de-colorized by soaking in ethanol (95%) for 15 min.

### 3.9. Samples Extraction for Colorimetric Analysis

For sample extraction, 1 g of leaves (corresponding to 5 leaves) was weighed and crushed in liquid nitrogen. The resulting powder was resuspended in 3 mL of 70% acetone, homogenized for 5 min by an Ultra-Turrax^®^ T-25 basic (IKA^®^-Werke GmbH & Co. KG, Staufen im Breisgau, Germany) and sonicated for about 20 min with Elma Transsonic T 460/H. A second step of homogenization was added to ensure the total lysis of plant material. The final mixture was centrifuged for 5 min at 4000 r.p.m. (1500 r.c.f.) (Eppendorf^®^ Microcentrifuge 5415D, Hamburg, Germany). Sample extraction was performed for colorimetric analysis using 3 plants. The experiment was conducted in triplicate. Results are expressed as the average of the three replicas ± the standard deviation.

### 3.10. Determination of Antioxidant Power

Antioxidant power was determined by the FRAP method (Ferric Ion Reducing Antioxidant Power) [46]. For each reaction, 20 µL of extract was added to fresh FRAP reagent composed of 2040 µL of 300 mM acetate buffer pH 3.6, 200 µL of 10 mM TPTZ (2,4,6-tripyridyl-s-triazine) and 200 µL of 20 mM ferric chloride (FeCl_3_). Samples were then incubated for 1 h at 37 °C. The antioxidant power was then measured by UV–Vis spectrophotometer (Double beam Perkin Elmer UV/Visible spectrophotometer, Waltham, MA, USA) sets at a maximum absorption of 593 nm. The absorbance value was interpolated on a standard curve of known solutions of ferrous sulfate. Values were expressed in mmol of ferrous chloride (Fe^2+^) equivalent per 100 g of leaves.

### 3.11. Determination of Phenolic Content

The content of total polyphenols was determined by the colorimetric assay Folin-Ciocalteu (F-C) [47]. For each reaction, 500 µL of extract were added to 3950 µL of distilled water, 250 µL of F-C reagent (Sigma Chemical, St. Louis, Missouri, USA), and 750 µL of a sodium carbonate saturated solution (Na_2_CO_3_). Samples were then incubated at 37 °C for 30 min. The absorbance was recorded by UV–Vis spectrophotometer (Double beam Perkin Elmer UV/Visible spectrophotometer, Waltham, Massachusetts, USA) at 795 nm. The spectrophotometric results were compared to a pre-made gallic acid standard (Sigma Chemical, St. Louis, Missouri, USA) curve. Total phenolic content was expressed as mg of gallic acid equivalents (GAE) per 100 g of leaves.

### 3.12. Determination of Flavonoid Content

The content of flavonoids was determined by the aluminum chloride method [48]. Briefly, 500 µL of extract were added to 1500 µL of 95% ethanol, 100 µL of 10% aluminum chloride solution (AlCl_3_), 100 µL of 1 M potassium acetate solution (CH_3_CO_2_K) and 2800 µL of distilled water. The obtained solution was left 30 min at room temperature. After incubation, absorbance was recorded by UV–Vis spectrophotometer (Double beam Perkin Elmer UV/Visible spectrophotometer, Waltham, Massachusetts, USA) set at 415 nm. Total flavonoid content was calculated in relation to a quercetin standard (Sigma Chemical, St. Louis, Missouri, USA) from a calibration curve and values were expressed as mg of quercetin equivalents (QeE) per 100 g of leaves.

### 3.13. Statistical Analysis

The normal distribution of data of the measured parameters was assessed by Kolmogorov–Smirnov and Shapiro–Wilk tests. Data of stem diameter were squared transformed while data of growth index, stomatal density, and lamina width were log10-transformed to achieve normality. For each genotype differences in the measured parameters (unless antioxidant power, phenolic and flavonoid content, see below) between the two treatments (control and drought stress) over time (days) were analyzed by two-way-factorial ANOVA followed by Tukey HSD test for multiple comparison of means. Data about the antioxidant power, phenolic and flavonoid content were tested as well by two-way factorial ANOVA followed by Tukey HSD test for the evaluation of differences between treatments and genotypes. All statistics were performed using STATISTICA with the α-error set at 0.05.

## 4. Conclusions

In conclusion, the analyses carried out in this study indicate that locally adapted tomato varieties exhibit a genotype-dependent response to drought. Our research is one of the few examples of studies on the effects of water stress on the vegetative growth of local Italian tomato varieties. Considering them collectively, our data show that the existence of a genotype-dependent response that may explain the different degree of tolerance to drought as observed among the varieties analyzed. We can therefore assume that the variety ‘Fragola’, classified as the most resistant genotype, upon perceiving the lack of water activates all possible mechanisms capable of preventing water loss: it reduces the number of stomata and, consequently, the water loss. In addition to this, we also observed a proper functioning of the photosynthetic apparatus (as demonstrated by the F_v_/F_m_ and PI values). The correct activity of photosynthetic apparatus also may explain why in ‘Fragola’ the production of ROS is limited and not sufficient to damage it. Moreover, the level of antioxidant molecules, associated with oxidative stress, is still comparable to well-irrigated plants. This would indicate that ‘Fragola’ has a very efficient enzymatic system of ROS scavenging that maintains the level of antioxidants comparable to well irrigated plants. This hypothesis is supported by evidence that enhancing the activities of antioxidant enzymes and/or accumulating low-Mr antioxidants by genetic manipulation increases tolerance of plant varieties to stresses through more efficient removal of ROS [49]. As a consequence of the correct functioning of the photosynthetic apparatus, all processes related to vegetative growth (e.g., plant height, leaf area, LaW, and LaL) are not compromised. On the contrary, the variety ‘Pisanello’ was proven to be the most susceptible: the variety attempts to implement a series of responses (such as variation of stomata density or anti-oxidant response); however, this variety is severely affected, suggesting that the mechanisms adopted are not sufficient to counteract the damage. This study therefore enables the discrimination of local tomato varieties exposed to water stress, suggesting the existence of a valuable reservoir of genetic biodiversity with more resistant tomato genotypes, and opening the way to research on the mechanisms of response to drought.

## Supplementary Materials

The following are available online at https://www.mdpi.com/2223-7747/8/9/336/s1, Figure S1: Images of regularly irrigated (control, CTRL) and drought-stressed (DS) tomato plants of the varieties ‘Costoluto Fiorentino’ and ‘Fragola’, respectively at time t_0_ (before treatment), t_1_ (half treatment) and t_2_ (end of treatment). Squares behind the plants have a side of 10 cm. Figure S2: Images of regularly irrigated (control, CTRL) and drought-stressed (DS) tomato plants of the varieties ‘Rosso di Pitigliano’ and ‘Canestrino di Lucca’, respectively at time t_0_ (before treatment), t_1_ (half treatment) and t_2_ (end of treatment). Squares behind the plants have a side of 10 cm. Figure S3: Images of regularly irrigated (control, CTRL) and drought-stressed (DS) tomato plants of the varieties ‘Pisanello’ and ‘Quarantino’, respectively at time t_0_ (before treatment), t_1_ (half treatment), and t_2_ (end of treatment). The double arrow in t_2_ indicates the difference in height between control and treated plants. Squares behind the plants have a side of 10 cm. Figure S4: Images of regularly irrigated (control, CTRL) and drought-stressed (DS) tomato plants of the commercial variety ‘Supersteak’, respectively at time t_0_ (before treatment), t_1_ (half treatment), and t_2_ (end of treatment). The double arrow in t_2_ indicates the difference in height between control and treated plants. Squares behind the plants have a side of 10 cm. Table S1: List of primer sequences used for genotyping.
