# Supplementary material for: Drought Stress Affects the Response of Italian Local Tomato (Solanum lycopersicum L.) Varieties in a Genotype-Dependent Manner
Source: Plants (Basel). 2019 Sep 7;8(9):336. doi: 10.3390/plants8090336 (PMC6783988; doi:10.3390/plants8090336)
Supplement: Supplementary file 1 [file plants-08-00336-s001.zip › Supplementary material/Table S1.docx]

| SSR Name | Core Motif | Primer Sequence (5’-3’) | Reference |
| --- | --- | --- | --- |
| LEat016 | (AT)₉ | fw: [6FAM]CCCAAATGCTATGCAATACAC  rv: AGTTCAGGATTGGTTTAAGGG | He et al. (2003) |
| LEcag003 | (CAG)₇ | fw: [HEX]CCGCCTCTTTCACTTGAAC  rv: CCAGCGATACGATTAGATACC | He et al. (2003) |
| LEaat007 | (AAT)₁₂ | fw: [6FAM]CAACAGCATAGTGGAGGAGG  rv: TACATTTCTCTCTCTCCCATGAG | He et al. (2003) |
| LEac001 | (AC)₉ | fw: [HEX]TGCCTTCCATCTAACCAATC  rv: CTGTGGCAAATATGTCCCTAAG | He et al. (2003) |
| LEatt001 | (ATT)₉ | fw: [6FAM]CCATTGTTCCATGCAGAAG  rv: CCAATGCTGATTTAATGCG | He et al. (2003) |
| LEcag001 | (CAG)₈ | fw: [HEX]ATGGTTCTTCATCAACAGCAG  rv: AGAAGTATTGAGCCAAGTCGG | He et al. (2003) |
| LEat002 | (AT)₉ | fw: [6FAM]ACTGCATTTCAGGTACATACTCTC  rv: ATAAACTCGTAGACCATACCCTC | He et al. (2003) |
| LEga003 | (GA)₂₀ | fw: [6FAM]TTCGGTTTATTCTGCCAACC  rv: GCCTGTAGGATTTTCGCCTA | He et al. (2003) |
| LEtat002 | (TAT)₁₂ | fw: [HEX]ACGCTTGGCTGCCTCGGA  rv: AACTTTATTATTGCCACGTAGTCATGA | He et al. (2003) |
| SSR70 | (AT)₂₀ | fw: [HEX]TTTAGGGTGTCTGGTGGGTCC  rv: GGAGTGCGCAGAGGATAGAG | Sardaro et al. (2013) |
| LEaat002 | (AAT)₁₂ | fw: [HEX]GCGAAGAAGATGAGTCTACAGCATAG  rv: CTCTCTCCCATGAGTTCTCCTCTTC | He et al. (2003) |
| SSR248 | (TA)₂₁ | fw: [6FAM]GCATTCGCTGTAGCTCGTTT  rv: GGGAGCTTCATCATAGTAACG | Sardaro et al. (2013) |
| SSR47 | (AT)₁₄ | fw: [HEX]TCCTCAAGAAATGAAGCTCTGA  rv: CCTTGGAGATAACAACCACAA | Sardaro et al. (2013) |
| SSR603 | (GAA)₈ | fw: [6FAM]GAAGGGACAATTCACAGAGTTTG  rv: CCTTCAACTTCACCACCACC | Sardaro et al. (2013) |
| TOM236 | (AT)₁₆ | fw: [6FAM]GTTTTTTCAACATCAAAGAGCT  rv: GGATAGGTTTCGTTAGTGAACT | Suliman-Pollatschek et al. (2002) |
| TOM210 | (ATA)₁₅ | fw: [HEX]CGTTGGATTACTGAGAGGTTTA  rv: ACAAAAATTCACCCACATCG | Suliman-Pollatschek et al. (2002) |

**Table S1.** List of primer sequences used for genotyping
