# Supplementary figures and images for: Drought Stress Affects the Response of Italian Local Tomato (Solanum lycopersicum L.) Varieties in a Genotype-Dependent Manner
Source: Plants (Basel). 2019 Sep 7;8(9):336. doi: 10.3390/plants8090336 (PMC6783988; doi:10.3390/plants8090336)

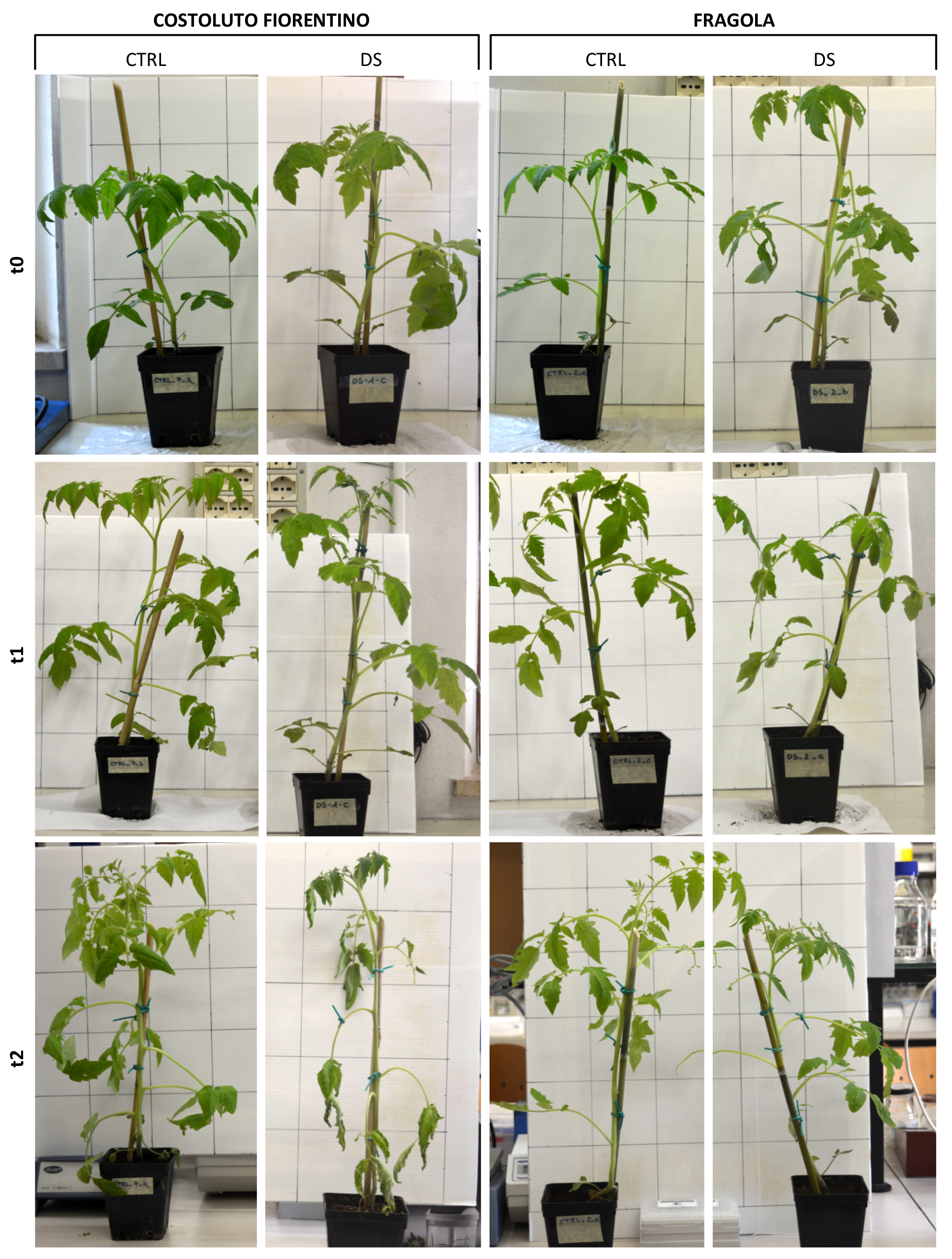

Supplement: Supplementary file 1 [file plants-08-00336-s001.zip › Supplementary material/figure S1.tif]

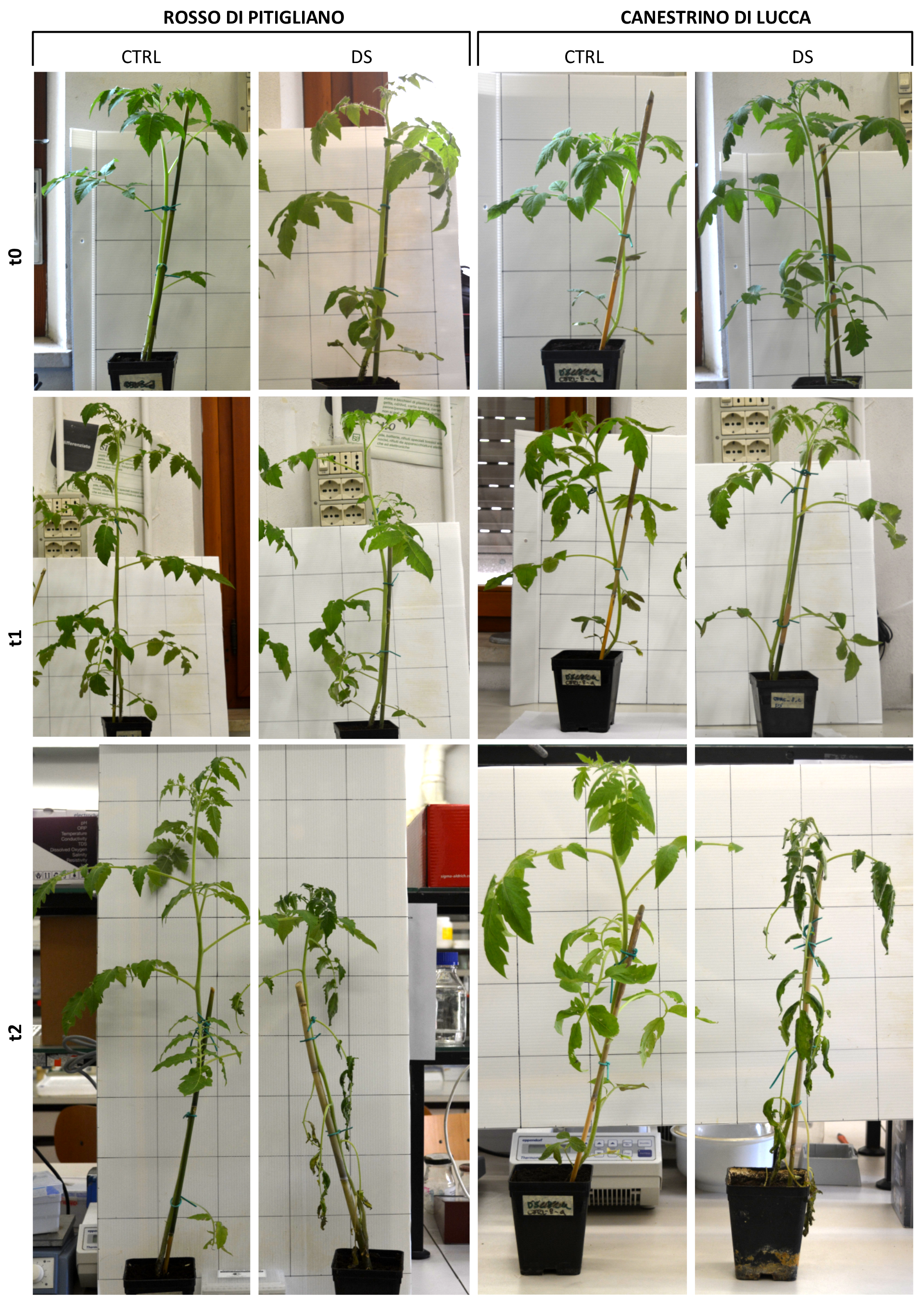

Supplement: Supplementary file 1 [file plants-08-00336-s001.zip › Supplementary material/figure S2.tif]

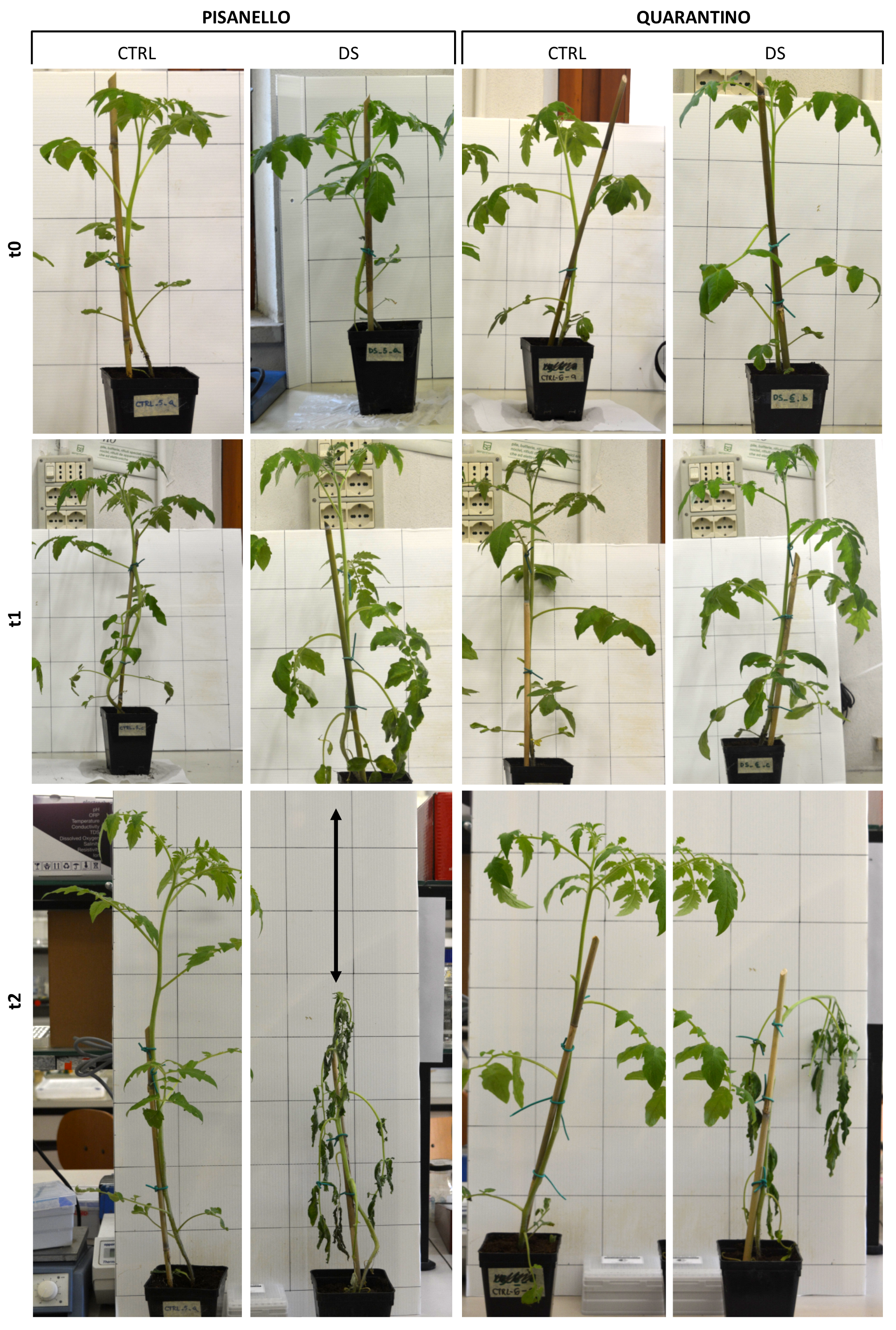

Supplement: Supplementary file 1 [file plants-08-00336-s001.zip › Supplementary material/figure S3.tif]

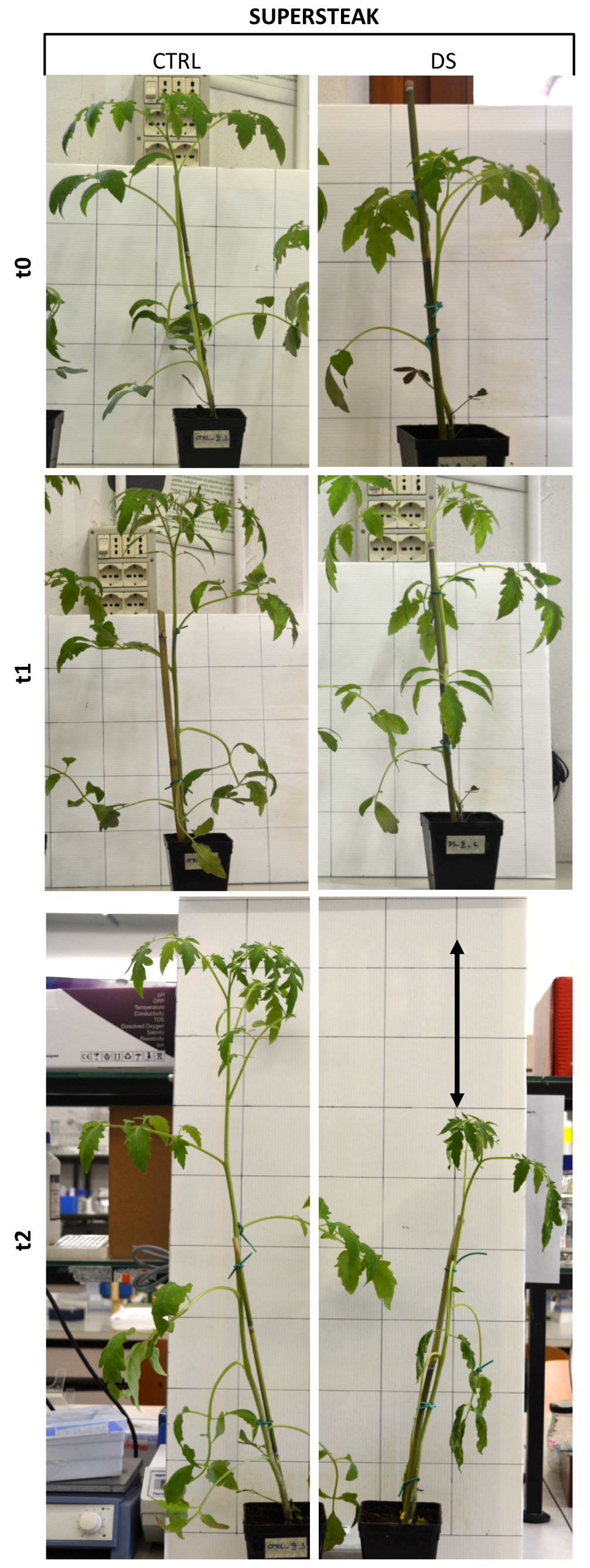

Supplement: Supplementary file 1 [file plants-08-00336-s001.zip › Supplementary material/figure S4.tif]
